# Extensive Backbone Cleavage Coverage of Intact Proteoforms in a Mass Range of 10–70 kDa by Integrating Electron, Collision, and Photon-Based Fragmentation Techniques during an Electrophoretic Time Scale

**DOI:** 10.1021/jasms.5c00384

**Published:** 2026-01-08

**Authors:** Qianjie Wang, Qianyi Wang, Rafael D. Melani, Quan Liu, Paul Nurmi, Liangliang Sun

**Affiliations:** † Department of Chemistry, 3078 Michigan State University, East Lansing, Michigan 48824, United States; ‡ Department of Biochemistry and Molecular Biology, Michigan State University, East Lansing, Michigan 48824, United States; § Thermo Fisher Scientific, San Jose, California 95134, United States; ∥ CMP Scientific Corp, Brooklyn, New York 11226, United States

## Abstract

Capillary zone electrophoresis (CZE)-tandem mass spectrometry (MS/MS) has been documented as a useful tool for top-down proteomics (TDP). However, CZE-MS/MS-based TDP typically has limited backbone cleavage coverage for identified proteoforms due to the use of traditional collision-based fragmentation methods (i.e., higher-energy collisional dissociation, HCD). Here, for the first time, we coupled CZE to an Orbitrap Ascend Tribrid mass spectrometer to investigate the performance of collision-, electron-, and photon-based fragmentation methods and their combinations for boosting the backbone cleavage coverage of proteoforms during the electrophoretic time scale using a standard protein mixture covering a mass range of about 10–70 kDa. CZE-MS achieved reproducible measurement of six proteins including three insulin-like growth factor (IGF) proteoforms with different modifications. Systematic investigations of HCD, electron-transfer dissociation (ETD), electron-transfer/HCD (EThcD), and ultraviolet photodissociation (UVPD) during CZE-MS/MS analysis revealed distinct yet complementary fragmentation characteristics. ETD, EThcD, and UVPD, in general, provided higher backbone cleavage coverage than HCD. The integration of HCD, ETD, EThcD, and UVPD data offered 67 and 98% sequence coverage for carbonic anhydrase (a 30 kDa protein) and thioredoxin (a 12 kDa protein), which is 158 and 100% higher than that produced by HCD alone. Adding internal fragments further boosted the backbone cleavage coverage substantially, for example, from 67 to 94% for 30 kDa carbonic anhydrase and from 21 to 82% for 50 kDa protein AG. The results demonstrate the capability of CZE-MS/MS with the integration of various fragmentation techniques for comprehensive characterization of proteoforms in a wide mass range.

## Graphical abstract

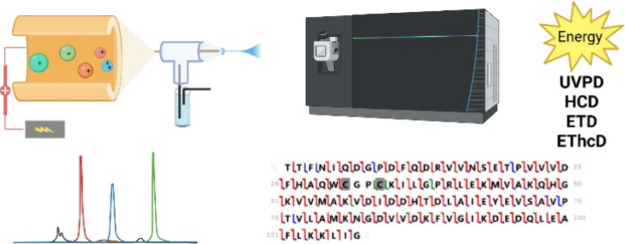

## 1 Introduction

Mass spectrometry (MS)-based top-down proteomics (TDP) enables comprehensive characterization of intact proteoforms, preserving labile and combinatorial post-translational modifications (PTMs), sequence variants, and truncations.^1,2^ With the focus on proteoform heterogeneity, TDP has been increasingly applied in biological research, disease studies, and therapeutic protein analysis.^3–5^ Achieving confident proteoform delineation in TDP depends on efficient front-end separation and effective gas-phase fragmentation.

Capillary zone electrophoresis (CZE) offers high-resolution separation of intact proteins based on electrophoretic mobility related to charge-to-size ratios.^6,7^ Proteoforms exhibiting subtle net charge or small mass differences—e.g., deamidation and phosphorylation—can be resolved prior to electrospray ionization (ESI). Compared to chromatographic methods (e.g., reversed-phase liquid chromatography, RPLC), the open tubular capillary in CZE minimizes the sample consumption and stationary-phase-related peak broadening, providing almost a million theoretical plates for intact proteoforms.^6,8,9^ CZE-MS has been widely recognized as a useful tool for TDP in various biomedical applications,^7,10–16^ because of its high separation efficiency, high sensitivity, and accurate prediction of electrophoretic mobility.^17–19^ CZE-MS has shown substantially higher sensitivity than RPLC-MS for proteoforms,^18^ which is invaluable for low-abundance proteoforms and proteoforms in mass-limited biological samples.

Fragmentation efficiency determines the extent of sequence coverage and the accuracy of PTM localization. Higher-energy collision dissociation (HCD) is widely used owing to its robustness and accessibility on b/y-type ions.^20^ Electron-based methods, such as electron transfer dissociation (ETD) and electron capture dissociation (ECD), provide complementary fragmentation by producing c/z-type ions.^21^ These approaches are particularly valuable for localizing labile PTMs, although their efficiency can decrease for larger proteins or precursors of low charge density.^22^ Ultraviolet photodissociation (UVPD) further expands the fragmentation space by generating multiple ion types (a/x, b/y, c/z) in a single spectrum.^23^ Under optimized conditions, UVPD can approach near-complete sequence coverage of monoclonal antibodies.^24^ UVPD remains one of the most powerful dissociation techniques for TDP. Recent advances in Orbitrap Tribrid mass spectrometers, including the Orbitrap Ascend, have enabled implementation of ETD, HCD, EThcD, and UVPD within a single platform.^25^ This integration facilitates systematic evaluation of multiple fragmentation strategies under uniform experimental conditions.^26^

Here, we report the first coupling of CZE to an Orbitrap Ascend Tribrid mass spectrometer for systematic evaluation of HCD, ETD, EThcD, and UVPD for fragmentation of intact proteoforms in a mass range of about 10–70 kDa during the CZE time scale. A standard protein mixture containing six proteins was used.

## 2 Methods

### 2.1 Materials and Chemicals

Pierce Intact Protein Standard Mix was purchased from Thermo Fisher Scientific (Cat#: A33526, Waltham, MA). Ammonium acetate (NH_4_OAc, 5 M) was purchased from Sigma-Aldrich (St. Louis, MO). LC/MS grade water, methanol, formic acid (FA), and acetic acid (AA) were purchased from Fisher Scientific (Pittsburgh, PA). Hydrofluoric acid (HF) and acrylamide were purchased from Acros Organics (Fair Lawn, NJ). The fused silica capillary (50 μm i.d., 360 μm o.d.) was purchased from Polymicro Technologies (Phoenix, AZ).

### 2.2 Sample Preparation

The intact Protein Standard Mix was prepared in 50 mM NH_4_OAc to achieve a final concentration of 0.5 mg/mL for the CZE-ESI-MS/MS analysis.

### 2.3 CZE-ESI-MS/MS Analysis

An EMASS-II CE-MS Ion Source commercialized by CMP Scientific (Brooklyn, NY)^27–29^ was used to couple CMP Scientific’s ECE-001 CE system to an Orbitrap Ascend Tribrid Mass Spectrometer (Thermo Fisher Scientific) equipped with IC, ETD, PTCR, and UVPD options.

An 80 cm long capillary (50 μm i.d., 360 μm o.d.) coated with linear polyacrylamide (LPA)^30^ with one end etched with hydrofluoric acid was used for separation. The LPA coating was prepared according to the literature.^11,31,32^ The background electrolyte (BGE) for CZE was 5% (v/v) AA (pH ∼ 2.4). The sheath buffer was 0.2% (v/v) FA containing 10% (v/v) methanol. High voltage (+30 kV) was applied for the CZE separation. For each CZE-MS/MS run, 50 nL of the sample was injected into the capillary by applying 100 mbar air pressure for 56 s based on Poiseuille’s law. The ESI emitters of the CE-MS interface were pulled from borosilicate glass capillaries (1.0 mm o.d., 0.75 mm i.d., 10 cm length) with a Sutter P-1000 flaming/brown micropipette puller. The opening size of the ESI emitters was 20–30 μm. The voltage for the ESI ranged from +2.0 to +2.3 kV.

For the mass spectrometer, all spectra were acquired in intact protein mode and low-pressure mode, and the method duration was set to 35 min. The ion transfer tube temperature and RF lens were set at 320 °C and 60%, respectively. For CZE-MS analysis, full MS scans were acquired with the detector type of Orbitrap at the 600–2000 *m*/*z* range with a resolution of 7500 (microscan number = 10) or 480,000 (microscan number = 2) at 200 *m*/*z*. Normalized AGC targets for MS were set at 200%, and the maximum injection time was set at auto. Source fragmentation was enabled with the energy at 15 V. The maximum injection time is 600 ms for a resolution of 7500 and 1019 ms for a resolution of 480,000.

To compare the fragmentation performance of HCD, UVPD (213 nm CryLaS laser), ETD, and EThcD, MS1 detector type was set as ion trap with the rapid scan rate at the scan range of 600–2000 *m*/*z*. One fragmentation method was assessed per CZE-MS/MS run. The maximum injection time was customized to 20 ms, and the normalized AGC target was 100%. The cycle time was set at 3 s. The microscan number was at 1. Source fragmentation was enabled with an energy of 15 V. For MS/MS experiments, the precursor ions were isolated by a quadrupole, and the isolation window was 4 *m*/*z* for the targeted fragmentation of the standard proteins. The multiplex ion function was enabled, and the maximum number of multiplex ions was 4. The number of MS2 isolations was 3 with 10 ms of MS2 CID activation time. The loop time was 3 s with a loop count of 20 and a loop control of 3. The MS/MS spectrum was acquired at a resolution of 120,000 at *m*/*z* 200 with two microscans. The detector type was set as orbitrap at the scan range of 600–2000 *m*/*z*. The normalized AGC target was 600% with a maximum injection time of 400 ms. The source fragmentation was enabled with an energy of 15 V. Table 1 shows the corresponding settings within different fragmentation options. Table 2 shows the list of targeted protein precursor ions, time ranges, and other details for targeted MS/MS.

**Table 1:** Fragmentation Conditions Applied for MS/MS Analysis of Intact Proteoforms^a^

fragmentation type	normalized collision energy (%)	activation time (ms)	supplemental activation collision energy (%)	terminal fragment types searched	internal fragment types searched
HCD	28, 32, 36	N/A	N/A	b and y	b, y, by
UVPD	N/A	5/10/25	N/A	UVPD9^a^	All fragments
ETD^a^	N/A	3, 6, auto	10	c and z	b, c, y, z, by, bz, cy, cz
EThcD	N/A	6	12, 15, 20	b, y, c, and z	b, c, y, z, by, bz, cy, cz

a Note: For all ETD options, ETD reagent target was set at 1.0E6, with a max ETD reagent injection time at 200 ms. N/A refers to no such settings within the corresponding fragmentation options. The UVPD 9 option was chosen in the ProSight Lite software for UVPD terminal fragment ions.

**Table 2:** Precursor Information for Targeted MS/MS Analysis during the CZE Run

**protein**	**avg mass (Da)**	**mono mass (Da)**	**time range (min)**	**precursor** *m*/*z*	***z***	**isolated window**(*m*/*z*)
EKFr	68,001.15	67,959.43	17–19.5	810.56	84	4
				820.31	83	
				840.52	81	
				872.8	78	
CA	28,981.29	28,963.69	18–20	853.4	34	4
				879.21	33	
				906.68	32	
IGF	9111.47	9105.35	19.6–21	1014	9	6
				1141	8	
				1303	7	
protein AG	50,459.74	50,429.85	20.8–21.7	1010	50	4
				1030	49	
				1052	48	
protein G	21,442.61	21,429.76	21.5–22.3	1022	21	4
				1129	19	
				1192	18	
thioredoxin	11,865.52	11,858.04	22.2–26	848	14	4
				913	13	
				989	12	

### 2.4 Data Analysis for Terminal Fragmentation Ions

Raw spectra of MS2 were deconvoluted with the Xtract algorithm (FreeStyle 1.8, Thermo Fisher Scientific). The *m*/*z* processing window was set at default. Neutral mass (“M”) was reported assuming proton adduction (H^+^, 1.00727663 Da). The charge-state search range was +5 to +60, with the minimum number of detected charge states set to 1. The analyzer-type parameter was set to OT (Orbitrap), and the isotope table was Protein. The relative abundance threshold was 0%. Negative-ion deconvolution was disabled. Unless otherwise noted, these parameters were used for all spectra deconvoluted in this study.

The deconvoluted fragments were imported into ProSight Lite.^33^ Fragmentation was assigned under the ETD, HCD, EThcD, and UVPD 9 mode (a, a^+^, b, c, x, x^+^, y, y^–^, and z) accordingly. The precursor mass type was set to monoisotopic, with mass mode defined as neutral (M). A fragmentation tolerance of 10 ppm was applied for all data sets, except for Protein AG, where a tolerance of 1 Da was used. Because Protein AG is large, the generated fragment ions can be large, and the monoisotopic mass of fragment ions can have much larger errors. Therefore, we used 1-Da mass tolerance for Protein AG.

### 2.5 Data Analysis for Internal Fragmentation Ions

Deconvoluted mass lists of carbonic anhydrase and streptococcus protein AG (Chimeric) by targeted HCD, UVPD, ETD, EThcD from the terminus fragmentation analysis were transformed to [M + H]^+^ masses and searched with ClipsMS against the protein sequence.^34^ The terminal fragment error (ppm) and the internal fragment error (ppm) were set to 10 and 1 ppm, respectively, with the smallest internal fragment size set at 5. No modifications were set, and the fragment types were searched based on the fragmentation options (Table 1).

## 3 Results and Discussion

### 3.1 Reproducible and High-Resolution Characterization of Proteoforms by CZE-MS

CZE-MS provided efficient and reproducible measurements of the intact protein mixture. The triplicate runs of CZE-MS under low-resolution (7500) and high-resolution (480,000) MS1 conditions are shown in Figure S1. The relative standard deviations (RSDs) of migration time of the proteins ranged from 0.09% to 0.38% for high-resolution MS1 runs and from 0.73 to 2.11% for low-resolution MS1 runs. The detailed protein information is listed in Table S1. Proteins spanning a broad mass range (9–68 kDa) were baseline-resolved within a ∼10 min migration window, reflecting their electrophoretic mobility differences driven by charge and size. Based on the accurate masses, the electrophoretic peaks were assigned to the six proteins, i.e., thioredoxin (Thio), Protein G, Protein AG, carbonic anhydrase (CA), *Escherichia coli* Exo Klenow Fragment (EKFr), and insulin-like growth factor (IGF), Figure 1A. The corresponding mass spectra of each protein are shown in Figure 1B–I. For EKFr (68 kDa), at high-resolution MS1 (480,000), no clear signal was observed due to the wide charge state distribution and complex isotopic envelopes; a clear signal was gained at low-resolution MS1 (7500), because isotopic peaks of each charge state were merged,^35^ Figure 1I.

**Figure 1:**
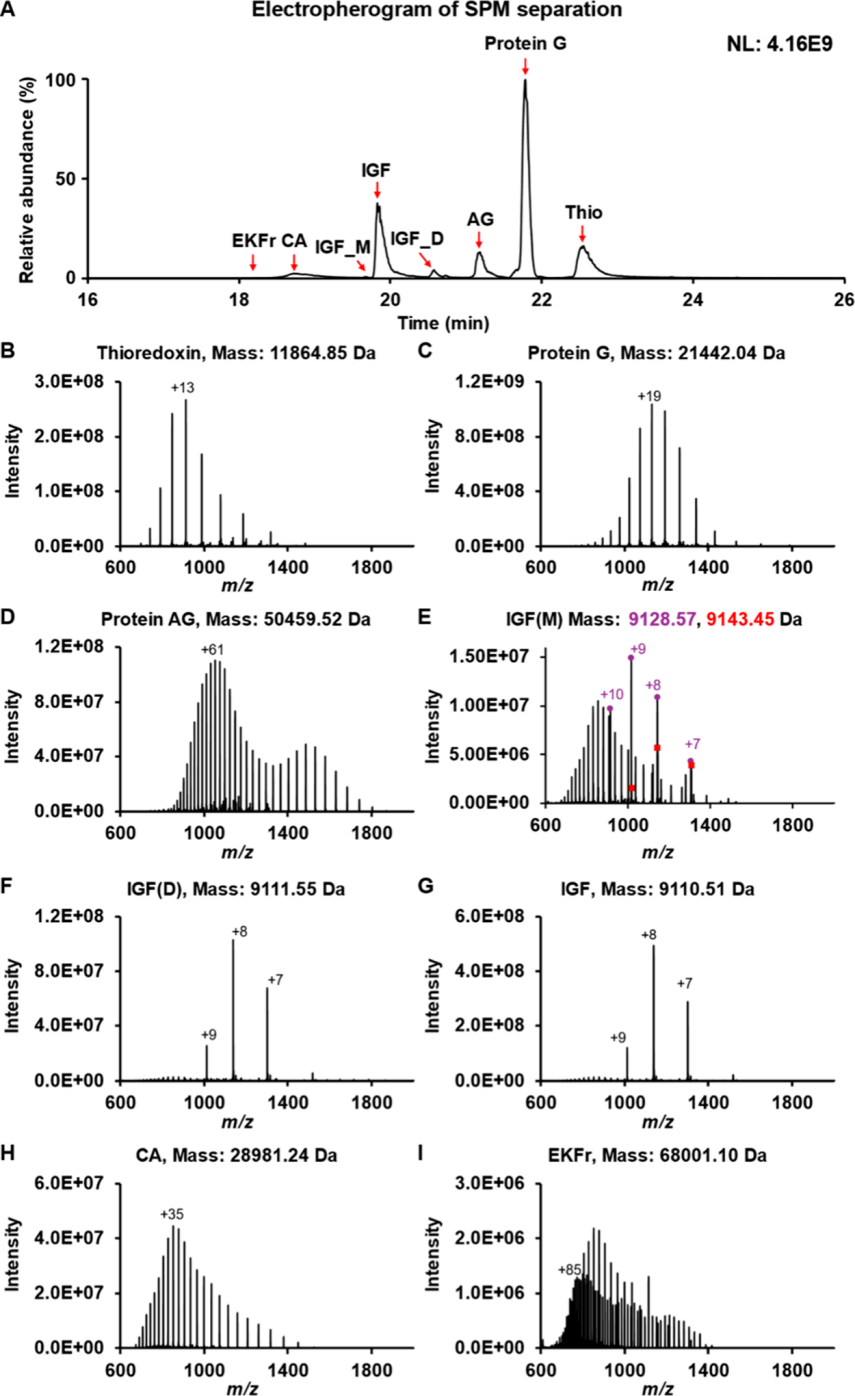
Separation electropherogram and mass spectra of the standard protein mixture (SPM) by CZE-ESI-MS at low resolution 7500. (A) Electropherogram of intact protein separation with annotated peaks for thioredoxin (Thio), Protein G, Protein AG, carbonic anhydrase (CA), *Escherichia coli* Exo Klenow Fragment (EKFr), insulin-like growth factor (IGF), its deamidated form (IGF_D), and an additional IGF-modified species IGF_M. (B–I) Representative deconvoluted mass spectra of separated proteins at a mass resolution of 7500 with their average masses.

For IGF, three peaks were resolved, Figure 1A. The most abundant peak (IGF) is the unmodified proteoform (average mass 9110.51 Da) with three disulfide bonds, Figure 1G. The peak IGF_D is the deamidated proteoforms with nearly 1-Da heavier than the IGF (average mass 9111.55 Da, Δm = +1.04 Da), Figure 1F. The data agrees well with that in our recently published work.^36^ The IGF_M peak contains two proteoforms that are 18.0035 and 31.9840 Da heavier than the unmodified proteoforms, Figure 1E. It is challenging to determine the exact source of these mass shifts based on the available information. One proteoform may result from one oxidation (+16 Da) with one fewer disulfide bond (+2 Da) relative to the main peak (IGF). The other proteoform may correspond to two oxidations (+32 Da). The separation of IGF, IGF_D, and IGF_M highlighted the advantage of CZE for separating proteoforms, especially those with PTMs that significantly affect their charge.

To determine the localization of modifications (i.e., deamidation) of IGF, we performed targeted MS/MS experiments under the conditions shown in Table 1. For ETD, the reaction times of 3 ms, 6 ms, and auto were tested, and a 10% HCD supplemental energy was applied to break the noncovalent interactions of ETD fragment ions for better measurement of c and z fragment ions. EThcD experiments combined ETD (6 ms) with HCD energies of 12, 15, and 20%. HCD-only dissociation was performed at normalized collision energies (NCE) of 28%, 32%, and 36%. UVPD was examined at activation periods of 5, 10, and 25 ms. These energy ladders provide dissociation power comparable to that across activation methods. The targeted precursor information is summarized in Table 2.

As shown in Figure 2A,B, the +0.9985 Da mass shift corresponding to Asn13 → Asp deamidation was determined. This mass shift was observed for y_73_ but absent in y_70_, pinpointing the deamidation site to Asn13 (N13). This assignment agrees well with the known spontaneous deamidation of Asn residues adjacent to glycine (N-G motif), forming a succinimide intermediate that converts to Asp, introducing an additional negative charge and reducing electrophoretic mobility under acidic conditions.^36–38^ The resulting conversion of an amide to a carboxyl group introduces one additional negative charge, decreasing electrophoretic mobility under acidic CZE conditions and producing the slightly delayed migration observed for IGF_D. The migration and characteristic fragmentation pattern together confirm that the deamidation occurred in solution rather than from in-source artifacts.^39^

**Figure 2:**
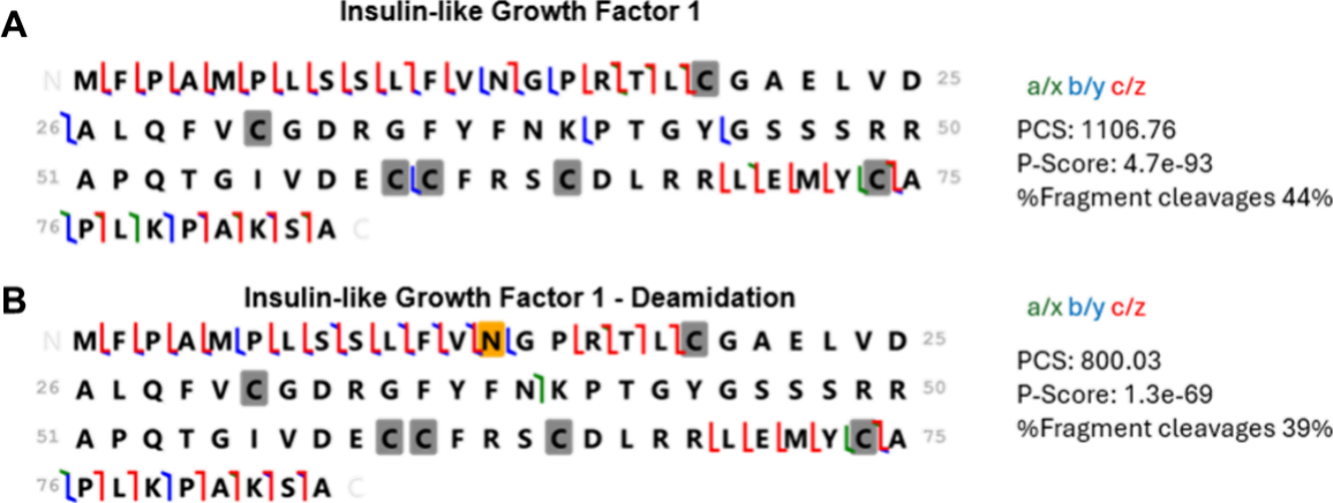
Fragmentation maps of IGF (A) and IGF_D (B) from the combinations of all fragmentation methods (HCD, ETD, EThcD, and UVPD). The deamidation site (N13) is highlighted in yellow. The cysteine residues marked in gray are those for disulfide bonds. PCS: proteoform characterization score. The data were generated using the ProSight Lite software.

The two higher-mass modified variants in IGF_M were coeluted earlier than the unmodified IGF despite their larger masses, indicating their higher number of positive charges in the solution. However, owing to the low signal intensity, coelution of two proteoforms, and the three disulfide bonds, the precise site and nature of modifications could not be assigned. Interestingly, the gas-phase charge-state distribution of the +18.0035-Da species shows higher charge states than the unmodified form, Figure 1E,G. Similarly, two larger proteoforms of Protein G migrated more slowly than did the unmodified Protein G peaks. The two larger proteoforms were +42.6 and +177.3 Da heavier than the unmodified one, and they could correspond to the acetylated Protein G and other modifications, which altered the protein’s electrophoretic mobility. The data here further highlights the value of CZE-MS for proteoforms compared to the typically used RPLC-MS, because CZE-MS can separate proteoforms with and without PTMs that significantly influence their charge, thereby helping confirm PTMs on proteoforms.^19^

### 3.2 Comparisons of Different Fragmentation Methods for Proteoform Cleavage

To evaluate the fragmentation performance of different dissociation techniques during the CZE time scale, we employed the targeted MS/MS approach with the details shown in Tables 1 and 2. The choice of fragmentation energies is based on our experience and literature data.^20,40–43^ The fragmentation maps of all proteoforms from various fragmentation techniques are shown in Figures S2–S7. Example MS/MS spectra with multiple annotated fragment ions are shown in Figures S8 and S9. We consider only the terminal fragment ions in this analysis. The summary of sequence coverage is listed in Table 3 and Table S2. Among the four activation methods evaluated (HCD, ETD, EThcD, and UVPD), ETD consistently produced similar or better cleavage coverage than HCD. For example, ETD produced 49% fragmentation coverage for Protein G (∼21 kDa), higher than HCD (32%); ETD generated higher fragmentation coverage than HCD for the 29-kDa CA (48% vs 26%). In theory, the combination of HCD and ETD (EThcD) should produce better fragmentation coverage than the HCD or ETD alone. Interestingly, in our data, EThcD data yielded less coverage than ETD for several proteoforms, Figures S2, S5, and S7. The reason could be the overfragmentation of proteoforms from EThcD. Furthermore, the increase in MS/MS spectrum complexity from EThcD made accurate mass deconvolution more difficult.

**Table 3:** Summary of the Fragmentation Coverage Data of Proteoforms under Different Fragmentation Conditions^a^

	**fragmentation coverage (%)**				
**protein**	**ETD**	**EThcD**	**HCD**	**UVPD**	**All**
CA	48	41	26	30	67
IGF	23	18	22	33	44
IGF_D	26	30	16	27	39
protein AG	9	7	9	7	21
protein G	49	59	32	28	73
thio	85	82	49	87	98

a Coverage values were calculated as the percentage of unique backbone cleavages assigned under each condition. ETD (10% HCD) was tested with 3 ms, 6 ms, and auto reaction times; EThcD was examined at 12, 15, and 20% supplemental activation energies. HCD was evaluated at 28, 32, and 36% normalized collision energies and UVPD at 5, 10, and 25 ms activation periods. Protein AG %fragmentation coverage was calculated using a 1 Da mass tolerance, while the others were calculated using a 10-ppm mass tolerance.

UVPD with a 213 nm laser produced better fragmentation coverage than HCD, ETD, and EThcD, under the conditions tested, for proteoforms with disulfide bonds, Figures S2 and S7. For example, UVPD got 33% cleavage coverage for IGF (three disulfide bonds), which is better than other techniques (18–23%), Figure S2. The tight structure of IGF makes it difficult to cleave by collision- and electron-based techniques, evidenced by no backbone cleavages for the middle region of the proteoform sequence, Figure S2. UVPD with high-enough energy can cleave the backbones in the middle area of the proteoform, resulting in higher fragmentation coverage, Figure S2. Interestingly, for the IGF-D, UVPD produced comparable fragmentation coverage with ETD and EThcD. For a slightly larger proteoform with one disulfide bond (thioredoxin), UVPD produced slightly better fragmentation coverage than EThcD and ETD (87% vs 82–85%) because of the looser structure compared to IGF, Figure S7. For large proteoforms without disulfide bonds (i.e., CA, protein G, and protein AG), UVPD generated fragmentation coverages lower than those of ETD or EThcD, Table 3 and Figures S4, S5, and S6. UVPD can fragment proteoforms in many pathways, producing highly complex MS/MS spectra, especially for large proteoforms. Averaging a large number of MS/MS spectra is typically employed to maintain sufficient S/N ratios of fragment ions. In our study, we only had a limited period for collecting UVPD data of these large proteoforms due to their narrow electrophoretic peaks (20–30 s), limiting the performance of UVPD.

Each dissociation method has distinct cleavage preferences, and the combination of various fragmentation techniques has enhanced the fragmentation coverage of all proteins studied here, Tables 3, S2, and Figures S2–S7. For example, combinations of fragment ions from all fragmentation techniques generated a 67% fragmentation coverage for CA (∼29 kDa), which is significantly higher than that achieved by any technique alone (26–48%). With the combination of all methods, CZE-MS/MS produced cleavage coverages of 44, 67, 98, and 73% for IGF, CA, thioredoxin, and protein G, respectively. For AG (∼50 kDa), the combined approach generated a fragmentation coverage of 21%, which is much lower than that of other proteins due to its large mass and the need for further method optimization.

### 3.3 Considering Internal Fragment Ions for Better Fragmentation Coverage

In addition to terminal ions, internal fragments have gained attention as an additional source of sequence information.^44,45^ These fragments can significantly improve coverage when they are included in analysis workflows. Tools such as ClipsMS have shown that internal fragments, particularly those generated by UVPD, extend sequence characterization beyond what is achieved with terminal ions alone.^34^

Protein backbone cleavages yield either (i) terminal fragment ions retaining the N-terminus (*a*, *b*, *c*) or C-terminus (*x*, *y*, *z*) of the precursor ions from single bond dissociation, or (ii) internal fragment ions formed by multiple cleavages, producing specific combinations (e.g., *ax*, *ay*, *az*, *bx*, *by*, *bz*, *cx*, *cy*, and *cz*), governed by the ion activation method and corresponding cleavage sites.^44,46–48^ Due to the large number of possible internal fragments that greatly exceeds that of terminal ions, their spectral assignment is inherently more ambiguous, particularly for larger proteins in TDP.^34^ The Loo lab has demonstrated that the use of the Clips-MS algorithm can enhance accurate internal fragment assignment in intact protein analysis by leveraging more fragment information from individual mass spectra.^34,46,49,50^ Here, we utilized Clips-MS for the analysis of internal fragment ions of CA (29 kDa) and Protein AG (50 kDa) fragmented by HCD, UVPD, ETD, and EThcD, and we compared the improvement of sequence coverage brought by the inclusion of internal fragments. To minimize the false positive misassignments, we applied a 1 ppm mass error tolerance for determining the internal fragment ions. The types of internal fragment ions searched for different fragmentation methods in this study are listed in Table 1.

For both cases, internal fragment analysis resulted in more extensive assignments of fragment ions compared to terminus-only fragment analysis, as shown in Figure 3A,B. The sequence coverage improvement of each fragmentation analysis for CA increased from 0.9% (ETD auto) to 12.4% (UVPD 25 ms), while Protein AG achieved a 3.3% (ETD auto, or UVPD 5 ms) to 26.9% (EThcD, 12%HCD) enhancement. Figures 3C and 3D show the sequence coverage heatmap from the combined data of all fragmentation techniques. The terminal fragment ions generated from these techniques tend to cover the N- and C-termini of the large proteins. These techniques produced a substantial amount of internal fragment ions covering the central regions of the large proteins. While some false positive internal ion assignments are inevitable, we minimized them with stringent criteria, and the observation here is not due to artifacts. Therefore, the inclusion of internal fragments can significantly enhance the fragmentation coverage. We achieved nearly full sequence coverage, i.e., 94% for CA and 82% for Protein AG, by combining all fragmentation techniques and terminal and internal fragment ions. Typically, obtaining in-depth fragmentation coverage of proteins larger than 30 kDa requires extensive spectral averaging through direct-infusion experiments.^51–53^ However, the results here demonstrate that the integration of CZE-MS/MS with multiple fragmentation strategies available on an Orbitrap Ascend Tribrid mass spectrometer, as well as the consideration of internal fragment analysis, is a practical approach for TDP with extensive fragmentation coverages of large proteoforms during the electrophoretic time scale.

**Figure 3:**
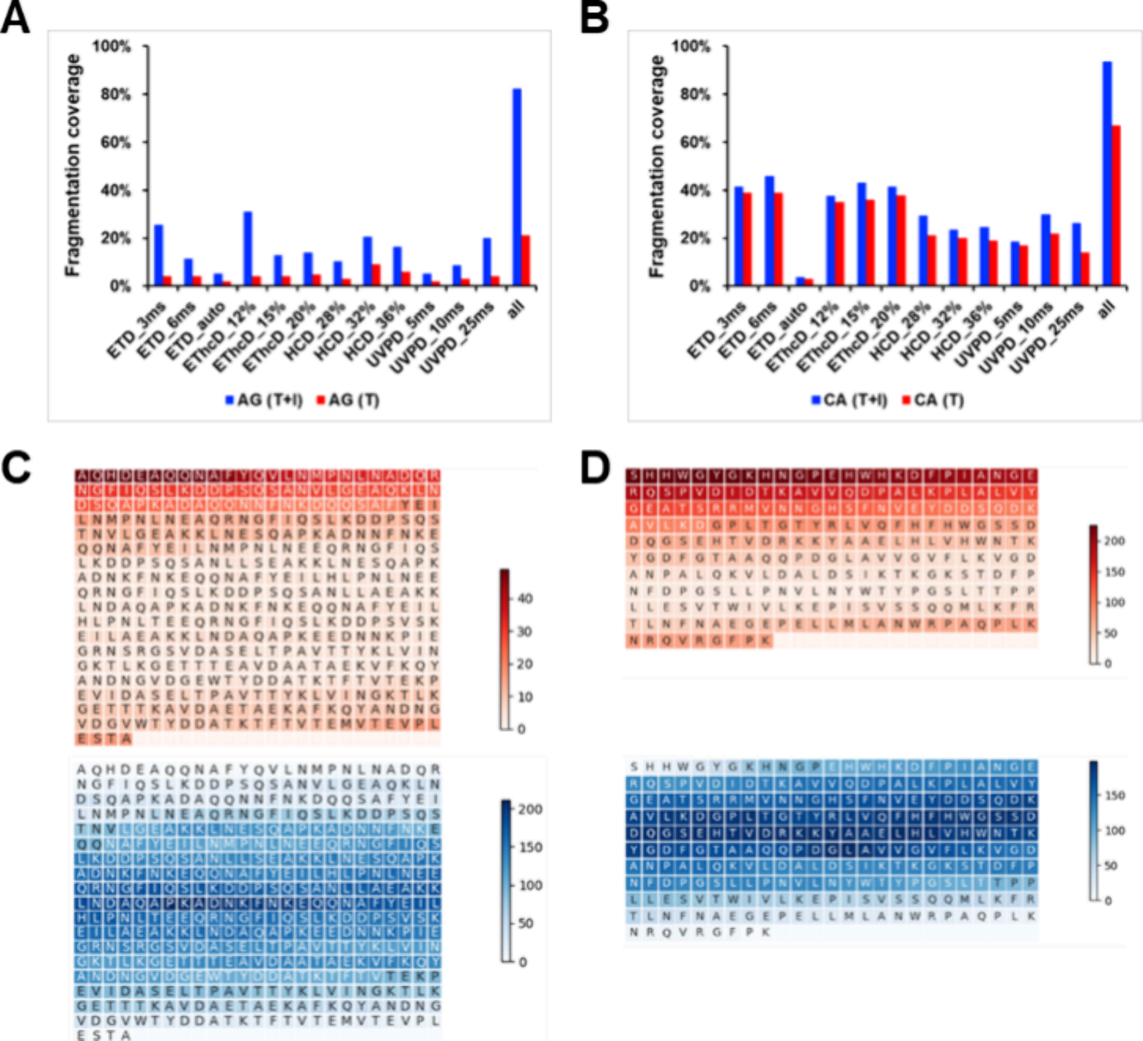
Representative fragmentation maps and sequence coverages of Protein AG (A, C) and carbonic anhydrase (CA) (B, D) from various fragmentation conditions. (A, B) Blue bars represent combined terminal and internal fragments (T + I); red bars represent terminal-only (T). “All” indicates cumulative coverage from all fragmentation methods. (C, D) Sequence coverage heatmaps illustrating terminal (top, red scale) and internal (bottom, blue scale) fragment ion assignments for Protein AG­(C) and CA­(D). Darker regions indicate more coverage. The fragment ions from all fragmentation techniques were combined and used here for the analysis in (C) and (D). The numbers on the red and blue color scales in (C) and (D) represent the number of fragment ions covering a specific region of the protein sequence.

## 4 Conclusions

This study demonstrates the power of CZE-MS/MS with multiple fragmentation techniques (i.e., ETD, HCD, EThcD, and UVPD) for the extensive characterization of proteoforms. CZE-MS achieved the reproducible separation and detection of intact proteins across a wide mass range (9–68 kDa) and resolved subtle proteoform variants (i.e., three IGF proteoforms). Under the conditions tested, UVPD (213 nm) produced better fragmentation coverage than ETD and EThcD for proteoforms containing disulfide bonds (i.e., IGF and thioredoxin). Incorporating internal fragment ions results in a nearly complete fragmentation coverage of large proteoforms (i.e., CA and Protein AG). Overall, we expect that the combination of efficient CZE separation and complementary gas-phase fragmentation will substantially advance the field of TDP for proteoform characterization. In our future work, we will employ this approach in a more complex biological system (i.e., whole cell lysates) to evaluate its performance.

## Supplementary Material



## Data Availability

The MS RAW files were deposited to the ProteomeXchange Consortium via PRIDE, with the data set identifier PXD070486.^54^

## Notes

Theoretical mass of proteins in the standard protein mixture, summary of fragmentation coverage data of proteoforms under different fragmentation conditions, electropherograms of triplicate CZE-MS runs under high-resolution and low-resolution MS1 conditions, detailed fragmentation maps of each protein under various dissociation conditions, and example annotated MS/MS spectra of CA (PDF)
