# Supplementary material for: Extensive Backbone Cleavage Coverage of Intact Proteoforms in a Mass Range of 10–70 kDa by Integrating Electron, Collision, and Photon-Based Fragmentation Techniques during an Electrophoretic Time Scale
Source: J Am Soc Mass Spectrom. 2026 Jan 8;37(2):505–13. doi: 10.1021/jasms.5c00384 (PMC12879928; doi:10.1021/jasms.5c00384)
Supplement: Supplementary file 1 [file js5c00384_si_001.pdf]

Supporting Information

Extensive backbone cleavage coverage of intact proteoforms in a mass range of 10-70 kDa by integrating electron, collision, and photon-based fragmentation techniques during an electrophoretic timescale

Qianjie Wang^{1,2#}, Qianyi Wang^{1,#}, Rafael D. Melani³, Quan Liu⁴, Paul Nurmi⁴, Liangliang Sun^{1,*}

¹Department of Chemistry, Michigan State University, East Lansing, MI 48824, USA.

²Department of Biochemistry and Molecular Biology, Michigan State University, East Lansing, MI 48824, USA.

³Thermo Fisher Scientific, San Jose, CA 95134, USA.

⁴CMP Scientific Corp, Brooklyn, NY 11226, USA.

#These authors contributed equally: Qianjie Wang, Qianyi Wang

*Corresponding author. Email: lsun@chemistry.msu.edu

Table S1. Composition and theoretical mass of the Pierce™ Intact Protein Standard Mix.

**Protein Name**	**Protein Accession**	**Theoretical Average Mass (Da)**	**Theoretical Monoisotopic Mass (Da)**
Escherichia coli Exo Klenow Fragment	P00582 (324-928)	68001.15	67959.42515
Bovine Carbonic Anhydrase II	P00921	28921.29	28963.6881
Human IGF-1 LR3	P05019 (40-118)	9111.47	9105.34872
Streptococcus Protein AG (chimeric)	P02976, P19909	50459.74	50429.84641
Streptococcus dysgalactiae Protein G	P06654 (223-413)	21442.61	21429.75915
Human Thioredoxin	Q99757(60-166)	11865.52	11858.04393

Table S2. Summary of fragmentation coverage data of proteoforms under different fragmentation conditions. *

Protein name	ETD(ms)			ETD<br>All	EThcD(NCE%)			EThcD<br>All	HCD(NCE%)			HCD<br>All	UVPD(ms)			UVPD<br>All	All
	3	6	auto		12	15	20		28	32	36		5	10	25		
CA	34	38	4	48	35	36	38	41	21	20	19	26	17	22	14	30	67
IGF	20	16	20	23	15	13	9	18	17	20	15	22	15	22	29	33	44
IGF_D	22	18	22	26	24	24	23	30	16	13	12	16	6	16	24	27	39
Protein AG*	4	4	2	9	4	4	5	7	3	9	6	9	2	3	4	7	21
Protein G	41	53	34	49	54	52	51	59	21	25	26	32	8	15	24	28	73
Thio	75	77	76	85	77	74	76	82	42	42	42	49	60	72	70	87	98

* The numbers listed in the table for each protein are the fragmentation coverage (%) under each specific fragmentation condition. Coverage values were calculated as the percentage of unique backbone cleavages assigned under each condition. ETD (10% HCD) was tested with 3 ms, 6 ms, and auto reaction times; EThcD was examined at 12%, 15%, and 20% supplemental activation energies. HCD was evaluated at 28%, 32%, and 36% normalized collision energies, and UVPD at 5, 10, and 25 ms activation periods.

* Protein AG % fragmentation coverage was calculated using a 1 Da mass tolerance, while the others were calculated using a 10-ppm mass tolerance.

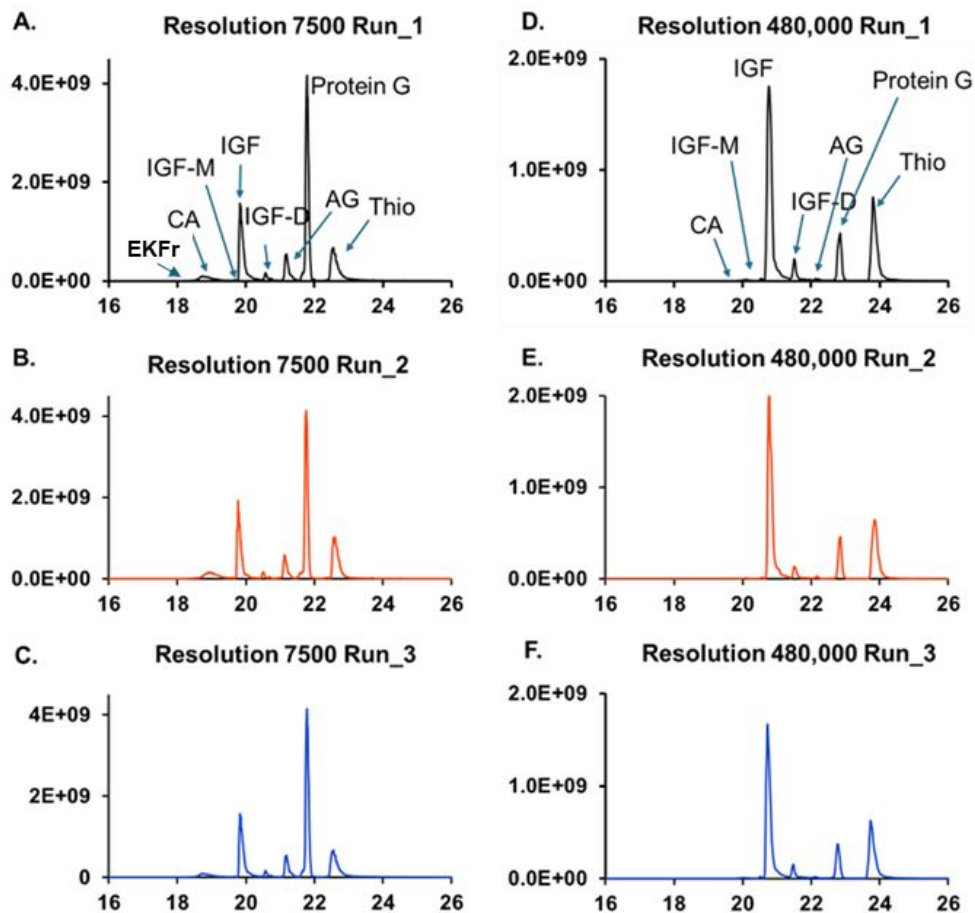

Figure S1. Reproducible CZE-MS separation of the intact standard protein mixture. (A–C) Representative electropherograms acquired at a mass resolution setting of 7,500 (at m/z 200), showing baseline separation and annotated peaks for thioredoxin (Thio), Protein G, Protein AG, carbonic anhydrase (CA), *Escherichia coli* Exo Klenow Fragment (EKFr), insulin-like growth factor (IGF), its deamidated form (IGF_D), and an additional IGF-modified species. (D–F) Electropherograms acquired at a higher resolution setting of 480,000 (at m/z 200).

Insulin-like growth factor (IGF)

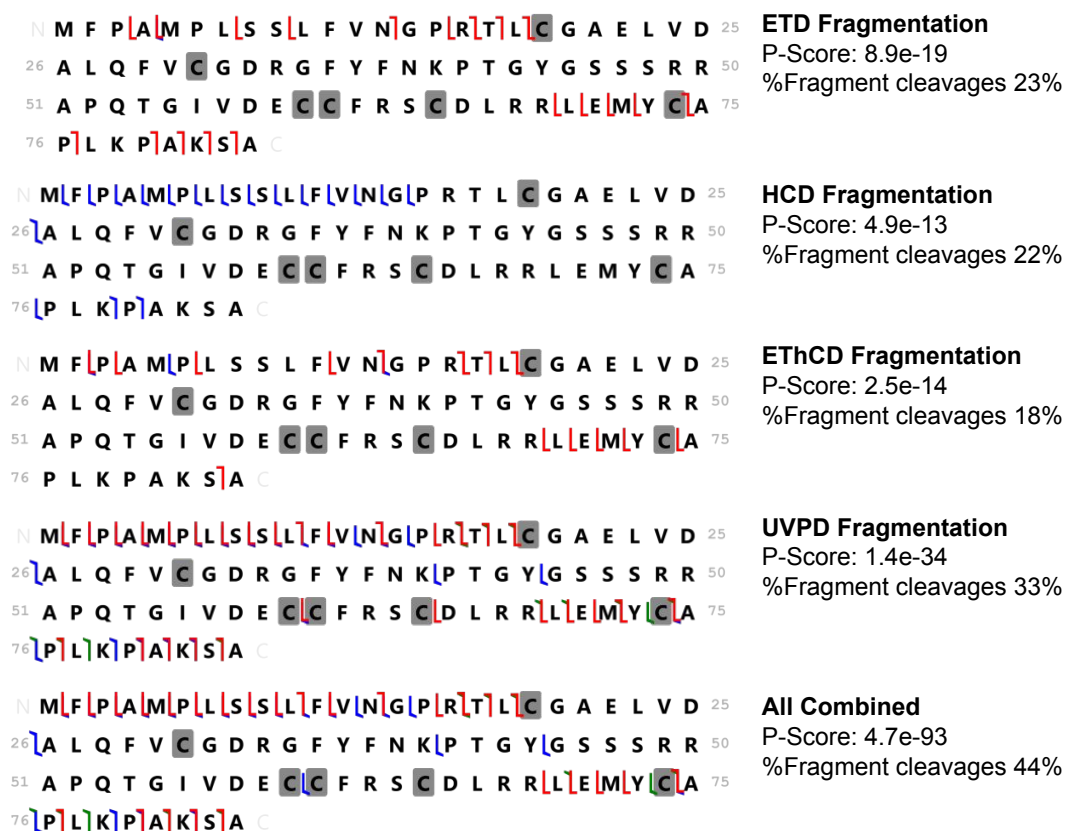

Figure S2. Fragmentation maps of insulin-like growth factor under different dissociation methods with a 10-ppm mass tolerance for fragment ion match. The data of each fragmentation technique is from the combined data of the three specific conditions listed in **Table 1**. The grey highlights the hydrogen loss on the cysteine due to the formation of disulfide bonds.

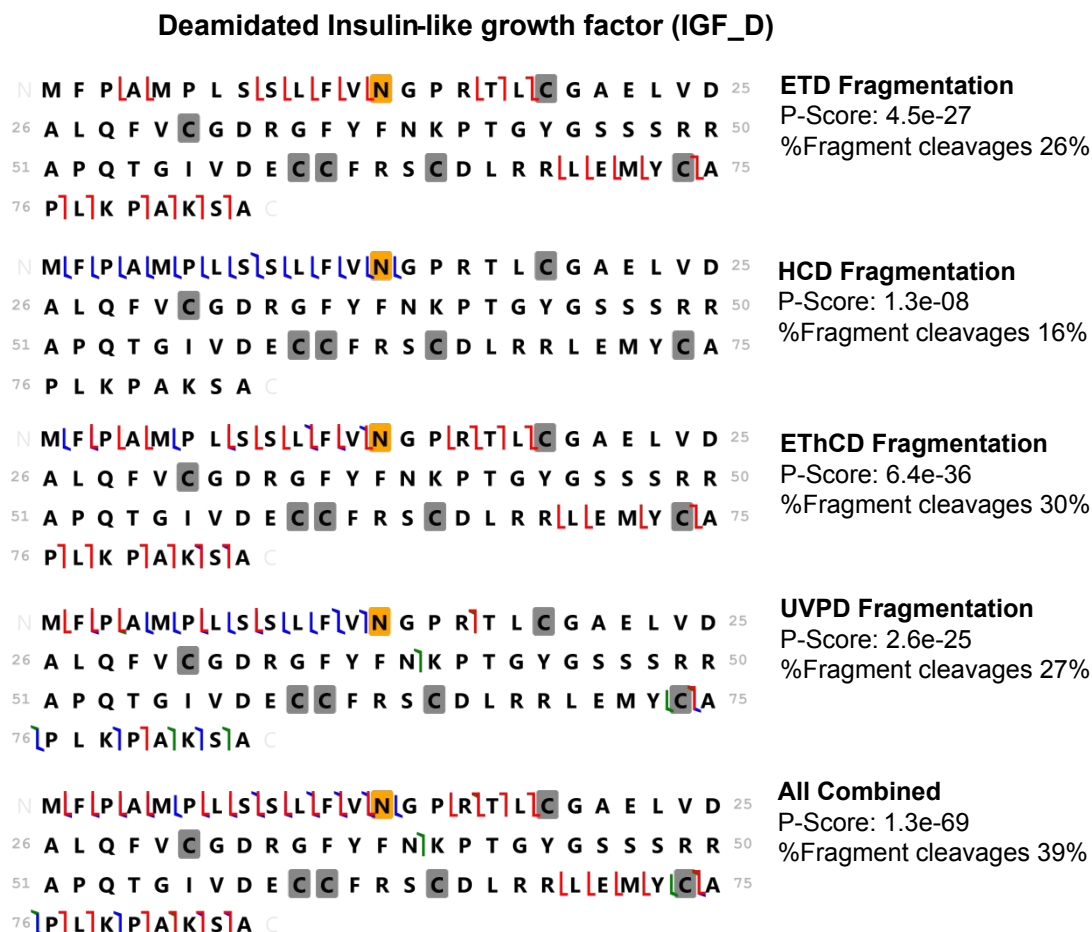

Figure S3. Fragmentation maps of deamidated insulin-like growth factor under different dissociation methods with a 10-ppm mass tolerance for fragment ion match. The data of each fragmentation technique is from the combined data of the three specific conditions listed in **Table 1**. The grey highlight is the hydrogen loss on the cysteine, and the orange highlight is the deamidation.

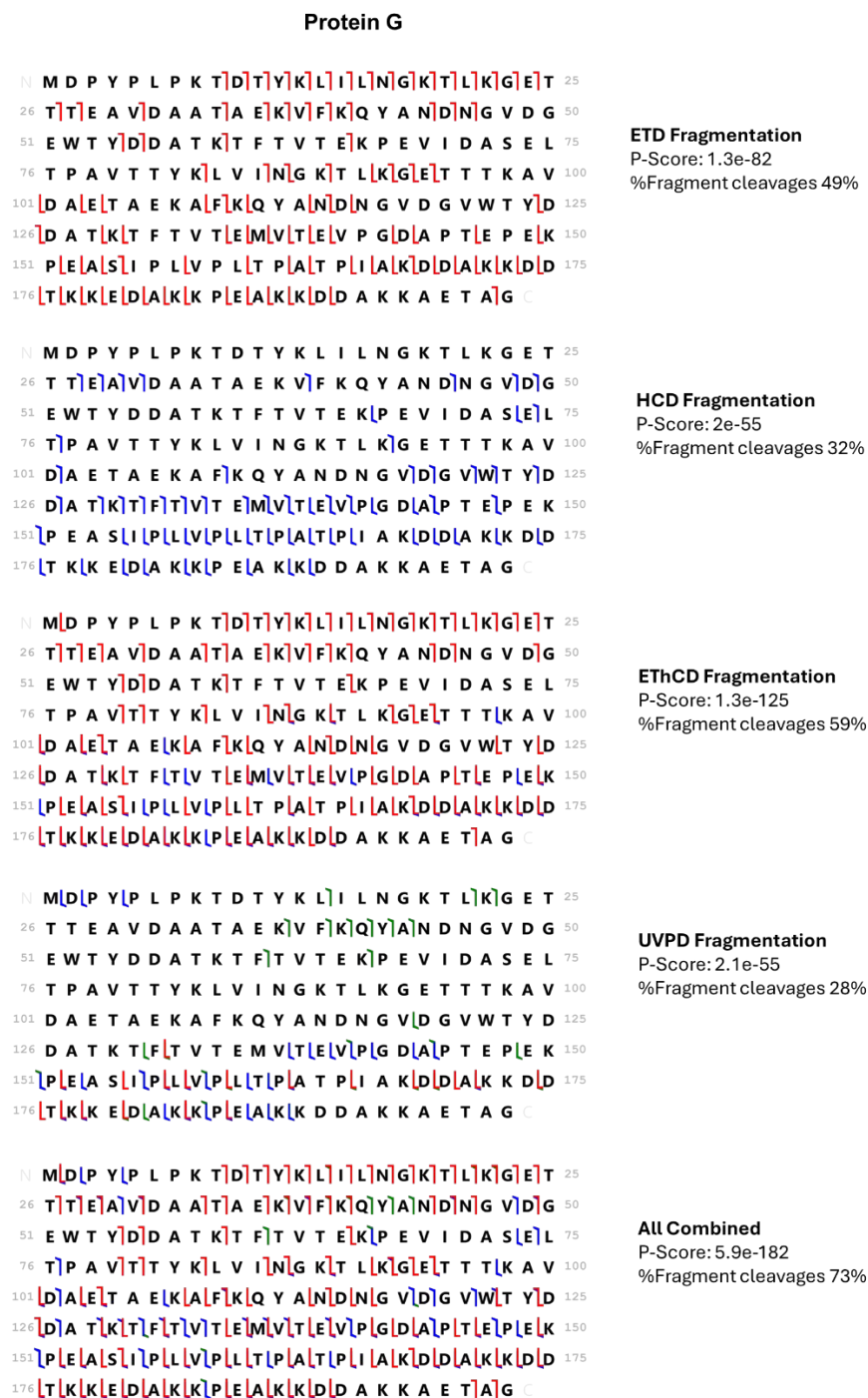

Figure S4. Fragmentation maps of Protein G under different dissociation methods with a 10-ppm mass tolerance for fragment ion match. The data of each fragmentation technique is from the combined data of the three specific conditions listed in **Table 1**.

Bovine Carbonic Anhydrase II

N S H H W G Y G K H N G P E H W H K D F P I A N G E 25
 26 R Q S P V D I D T K A V V Q D P A L K P L A L V Y 50
 51 G E A T S R R M V N N G H S F N V E Y D D S Q D K 75
 76 A V L K D G P L T G T Y R L V Q F H F H W G S S D 100
 101 D Q G S E H T V D R K K Y A A E L H L V H W N T K 125
 126 Y G D F G T A A Q P D G L A V V G V F L K V G D 150
 151 A N P A L Q K V L D A L D S I K T K G K S T D F P 175
 176 N F D P G S L L P N V L N Y W T Y P G S L T T P P 200
 201 L L E S V T W I V L K E P I S V S S Q Q M L K F R 225
 226 T L N F N A E G E P E L L M L A N W R P A Q P L K 250
 251 N R Q V R G F P K C

N S H H W G Y G K H N G P E H W H K D F P I A N G E 25
 26 R Q S P V D I D T K A V V Q D P A L K P L A L V Y 50
 51 G E A T S R R M V N N G H S F N V E Y D D S Q D K 75
 76 A V L K D G P L T G T Y R L V Q F H F H W G S S D 100
 101 D Q G S E H T V D R K K Y A A E L H L V H W N T K 125
 126 Y G D F G T A A Q Q P D G L A V V G V F L K V G D 150
 151 A N P A L Q K V L D A L D S I K T K G K S T D F P 175
 176 N F D P G S L L P N V L N Y W T Y P G S L T T P P 200
 201 L L E S V T W I V L K E P I S V S S Q Q M L K F R 225
 226 T L N F N A E G E P E L L M L A N W R P A Q P L K 250
 251 N R Q V R G F P K C

N S H H W G Y G K H N G P E H W H K D F P I A N G E 25
 26 R Q S P V D I D T K A V V Q D P A L K P L A L V Y 50
 51 G E A T S R R M V N N G H S F N V E Y D D S Q D K 75
 76 A V L K D G P L T G T Y R L V Q F H F H W G S S D 100
 101 D Q G S E H T V D R K K Y A A E L H L V H W N T K 125
 126 Y G D F G T A A Q Q P D G L A V V G V F L K V G D 150
 151 A N P A L Q K V L D A L D S I K T K G K S T D F P 175
 176 N F D P G S L L P N V L N Y W T Y P G S L T T P P 200
 201 L L E S V T W I V L K E P I S V S S Q Q M L K F R 225
 226 T L N F N A E G E P E L L M L A N W R P A Q P L K 250
 251 N R Q V R G F P K C

N S H H W G Y G K H N G P E H W H K D F P I A N G E 25
 26 R Q S P V D I D T K A V V Q D P A L K P L A L V Y 50
 51 G E A T S R R M V N N G H S F N V E Y D D S Q D K 75
 76 A V L K D G P L T G T Y R L V Q F H F H W G S S D 100
 101 D Q G S E H T V D R K K Y A A E L H L V H W N T K 125
 126 Y G D F G T A A Q Q P D G L A V V G V F L K V G D 150
 151 A N P A L Q K V L D A L D S I K T K G K S T D F P 175
 176 N F D P G S L L P N V L N Y W T Y P G S L T T P P 200
 201 L L E S V T W I V L K E P I S V S S Q Q M L K F R 225
 226 T L N F N A E G E P E L L M L A N W R P A Q P L K 250
 251 N R Q V R G F P K C

N S H H W G Y G K H N G P E H W H K D F P I A N G E 25
 26 R Q S P V D I D T K A V V Q D P A L K P L A L V Y 50
 51 G E A T S R R M V N N G H S F N V E Y D D S Q D K 75
 76 A V L K D G P L T G T Y R L V Q F H F H W G S S D 100
 101 D Q G S E H T V D R K K Y A A E L H L V H W N T K 125
 126 Y G D F G T A A Q Q P D G L A V V G V F L K V G D 150
 151 A N P A L Q K V L D A L D S I K T K G K S T D F P 175
 176 N F D P G S L L P N V L N Y W T Y P G S L T T P P 200
 201 L L E S V T W I V L K E P I S V S S Q Q M L K F R 225
 226 T L N F N A E G E P E L L M L A N W R P A Q P L K 250
 251 N R Q V R G F P K C

N S H H W G Y G K H N G P E H W H K D F P I A N G E 25
 26 R Q S P V D I D T K A V V Q D P A L K P L A L V Y 50
 51 G E A T S R R M V N N G H S F N V E Y D D S Q D K 75
 76 A V L K D G P L T G T Y R L V Q F H F H W G S S D 100
 101 D Q G S E H T V D R K K Y A A E L H L V H W N T K 125
 126 Y G D F G T A A Q Q P D G L A V V G V F L K V G D 150
 151 A N P A L Q K V L D A L D S I K T K G K S T D F P 175
 176 N F D P G S L L P N V L N Y W T Y P G S L T T P P 200
 201 L L E S V T W I V L K E P I S V S S Q Q M L K F R 225
 226 T L N F N A E G E P E L L M L A N W R P A Q P L K 250
 251 N R Q V R G F P K C

ETD Fragmentation

P-Score: 2.9e-118

%Fragment cleavages 48%

HCD Fragmentation

P-Score: 3.4e-34

%Fragment cleavages 26%

EthCD Fragmentation

P-Score: 1.4e-126

%Fragment cleavages 41%

UVPD Fragmentation

P-Score: 6.4e-53

%Fragment cleavages 30%

All Combined

P-Score: 5.9e-165

%Fragment cleavages 67%

Figure S5. Fragmentation maps of CA under different dissociation methods with a 10-ppm mass tolerance for fragment ion match. The data of each fragmentation technique is from the combined data of the three specific conditions listed in **Table 1**.

Protein AG

ETD Fragmentation

P-Score: 0.36

%Fragment cleavages 9%

```

1  A Q H D E A Q Q N A F Y Q V L N M P N L N A D Q R 25
24 N G F I Q S L K D P S Q S A N V L G E A Q K L N 50
51 D S Q A P K A D A Q Q N N F N K D Q Q S A F Y E I 75
76 L N M P N L N E A Q R N G F I Q S L K D D P S Q S 100
101 T N V L G E A K K L N E S Q A P K A D N N F N K E 125
126 Q Q N A F Y E I L N M P N L N E E Q R N G F I Q S 150
151 L K D D P S Q S A N L L S E A K K L N E S Q A P K 175
176 A D N K F N K E Q Q N A F Y E I L H L P N L N E E 200
201 Q R N G F I Q S L K D D P S Q S A N L L A E A K K 225
226 L N D A Q A P K A D N K F N K E Q Q N A F Y E I L 250
251 H L P N L T E E Q R N G F I Q S L K D D P S V S K 275
276 E I L A E A K K L N D A Q A P K E E D N N K P I E 300
301 G R N S R G S V D A S E L T P A V T T Y K L V I N 325
326 G K T L K G E T T T E A V D A A T A E K V F K Q Y 350
351 A N D N G V D G E W T Y D D A T K T F T V T E K P 375
376 E V I D A S E L T P A V T T Y K L V I N G K L T L K 400
401 G E T T T K A V D A E T A E K A F K Q Y A N D N G 425
426 V D G V W T Y D D A T K L T F L T V T E M V T E V P L 450
451 E S T A

```

HCD Fragmentation

P-Score: 0.0048

%Fragment cleavages 9%

```

1  A Q H D E A Q Q N A F Y Q V L N M P N L N A D Q R 25
24 N G F I Q S L K D D P S Q S A N V L G E A Q K L N 50
51 D S Q A P K A D A Q Q N N F N K D Q Q S A F Y E I 75
76 L N M P N L N E A Q R N G F I Q S L K D D P S Q S 100
101 T N V L G E A K K L N E S Q A P K A D N N F N K E 125
126 Q Q N A F Y E I L N M P N L N E E Q R N G F I Q S 150
151 L K D D P S Q S A N L L S E A K K L N E S Q A P K 175
176 A D N K F N K E Q Q N A F Y E I L H L P N L N E E 200
201 Q R N G F I Q S L K D D P S Q S A N L L A E A K K 225
226 L N D A Q A P K A D N K F N K E Q Q N A F Y E I L 250
251 H L P N L T E E Q R N G F I Q S L K D D P S V S K 275
276 E I L A E A K K L N D A Q A P K E E D N N K P I E 300
301 G R N S R G S V D A S E L T P A V T T Y K L V I N 325
326 G K T L K G E T T T E A V D A A T A E K V F K Q Y 350
351 A N D N G V D G E W T Y D D A T K T F T V T E K P 375
376 E V I D A S E L T P A V T T Y K L V I N G K T L K 400
401 G E T T T K A V D A E T A E K A F K Q Y A N D N G 425
426 V D G V W T Y D D A T K T F T V T E M V T E V P L 450
451 E S T A

```

UVPD Fragmentation

P-Score: 1.1e-06

%Fragment cleavages 7%

```

1  A Q H D E A Q Q N A F Y Q V L N M P N L N A D Q R 25
24 N G F I Q S L K D D P S Q S A N V L G E A Q K L N 50
51 D S Q A P K A D A Q Q N N F N K D Q Q S A F Y E I 75
76 L N M P N L N E A Q R N G F I Q S L K D D P S Q S 100
101 T N V L G E A K K L N E S Q A P K A D N N F N K E 125
126 Q Q N A F Y E I L N M P N L N E E Q R N G F I Q S 150
151 L K D D P S Q S A N L L S E A K K L N E S Q A P K 175
176 A D N K F N K E Q Q N A F Y E I L H L P N L N E E 200
201 Q R N G F I Q S L K D D P S Q S A N L L A E A K K 225
226 L N D A Q A P K A D N K F N K E Q Q N A F Y E I L 250
251 H L P N L T E E Q R N G F I Q S L K D D P S V S K 275
276 E I L A E A K K L N D A Q A P K E E D N N K P I E 300
301 G R N S R G S V D A S E L T P A V T T Y K L V I N 325
326 G K T L K G E T T T E A V D A A T A E K V F K Q Y 350
351 A N D N G V D G E W T Y D D A T K T F T V T E K P 375
376 E V I D A S E L T P A V T T Y K L V I N G K T L K 400
401 G E T T T K A V D A E T A E K A F K Q Y A N D N G 425
426 V D G V W T Y D D A T K T F T V T E M V T E V P L 450
451 E S T A

```

EThcD Fragmentation

P-Score: 0.23

%Fragment cleavages 7%

```

1  A Q H D E A Q Q N A F Y Q V L N M P N L N A D Q R 25
24 N G F I Q S L K D D P S Q S A N V L G E A Q K L N 50
51 D S Q A P K A D A Q Q N N F N K D Q Q S A F Y E I 75
76 L N M P N L N E A Q R N G F I Q S L K D D P S Q S 100
101 T N V L G E A K K L N E S Q A P K A D N N F N K E 125
126 Q Q N A F Y E I L N M P N L N E E Q R N G F I Q S 150
151 L K D D P S Q S A N L L S E A K K L N E S Q A P K 175
176 A D N K F N K E Q Q N A F Y E I L H L P N L N E E 200
201 Q R N G F I Q S L K D D P S Q S A N L L A E A K K 225
226 L N D A Q A P K A D N K F N K E Q Q N A F Y E I L 250
251 H L P N L T E E Q R N G F I Q S L K D D P S V S K 275
276 E I L A E A K K L N D A Q A P K E E D N N K P I E 300
301 G R N S R G S V D A S E L T P A V T T Y K L V I N 325
326 G K T L K G E T T T E A V D A A T A E K V F K Q Y 350
351 A N D N G V D G E W T Y D D A T K T F T V T E K P 375
376 E V I D A S E L T P A V T T Y K L V I N G K T L K 400
401 G E T T T K A V D A E T A E K A F K Q Y A N D N G 425
426 V D G V W T Y D D A T K L T F L T V T E M V T E V P L 450
451 E S T A

```

All Combined

P-Score: 3.5e-33

%Fragment cleavages: 21%

```

1  A Q H D E A Q Q N A F Y Q V L N M P N L N A D Q R 25
24 N G F I Q S L K D D P S Q S A N V L G E A Q K L N 50
51 D S Q A P K A D A Q Q N N F N K D Q Q S A F Y E I 75
76 L N M P N L N E A Q R N G F I Q S L K D D P S Q S 100
101 T N V L G E A K K L N E S Q A P K A D N N F N K E 125
126 Q Q N A F Y E I L N M P N L N E E Q R N G F I Q S 150
151 L K D D P S Q S A N L L S E A K K L N E S Q A P K 175
176 A D N K F N K E Q Q N A F Y E I L H L P N L N E E 200
201 Q R N G F I Q S L K D D P S Q S A N L L A E A K K 225
226 L N D A Q A P K A D N K F N K E Q Q N A F Y E I L 250
251 H L P N L T E E Q R N G F I Q S L K D D P S V S K 275
276 E I L A E A K K L N D A Q A P K E E D N N K P I E 300
301 G R N S R G S V D A S E L T P A V T T Y K L V I N 325
326 G K T L K G E T T T E A V D A A T A E K V F K Q Y 350
351 A N D N G V D G E W T Y D D A T K T F T V T E K P 375
376 E V I D A S E L T P A V T T Y K L V I N G K T L K 400
401 G E T T T K A V D A E T A E K A F K Q Y A N D N G 425
426 V D G V W T Y D D A T K L T F L T V T E M V T E V P L 450
451 E S T A

```

Figure S6. Fragmentation maps of Protein AG under different dissociation methods with a 1-Da mass tolerance for fragment ion match. The data of each fragmentation technique is from the combined data of the three specific conditions listed in **Table 1**.

Thioredoxin

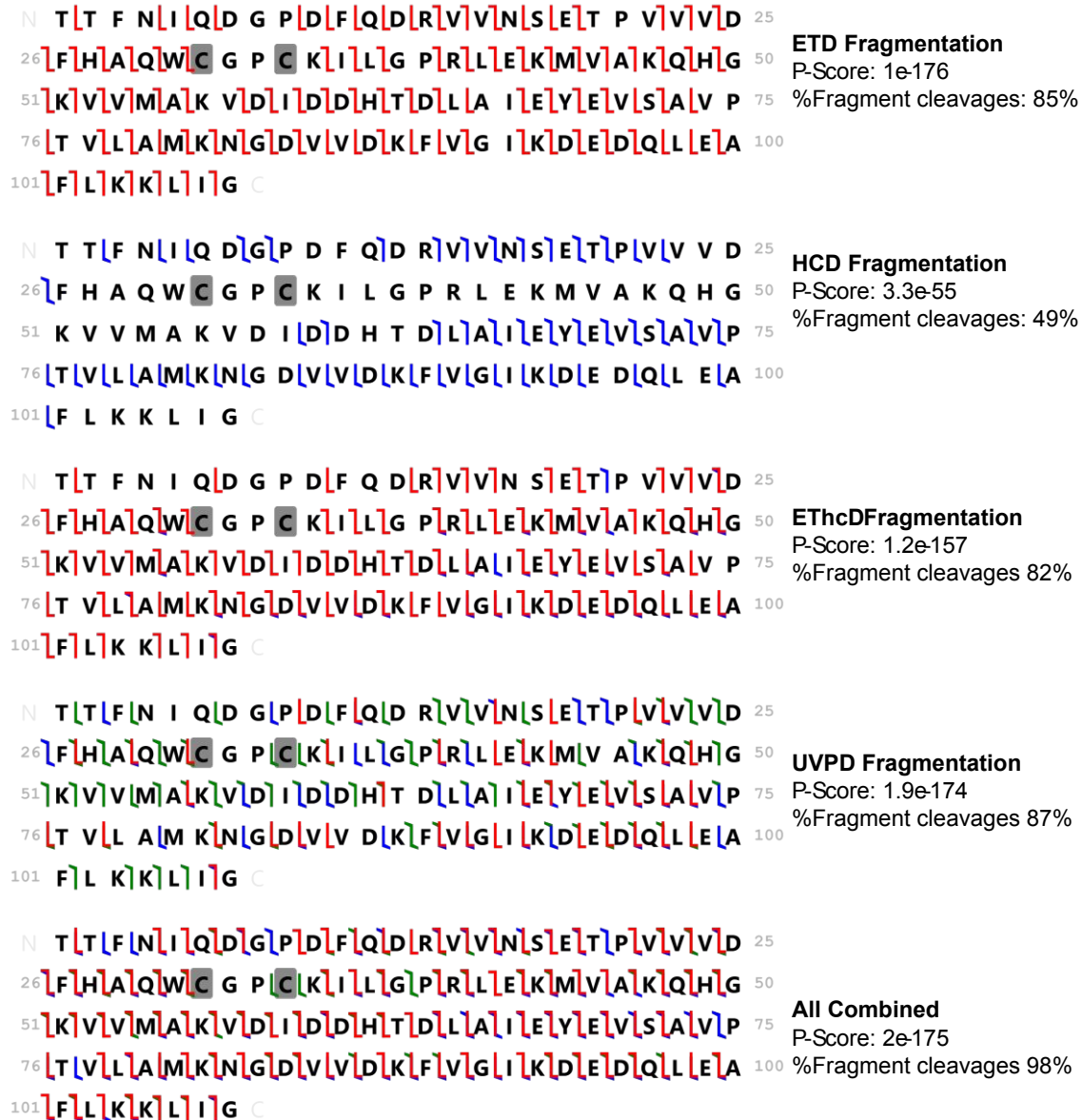

Figure S7. Fragmentation maps of Thioredoxin under different dissociation methods with a 10-ppm mass tolerance for fragment ion match. The data of each fragmentation technique is from the combined data of the three specific conditions listed in **Table 1**. The grey highlight is the hydrogen loss on the cysteine for the disulfide bond.

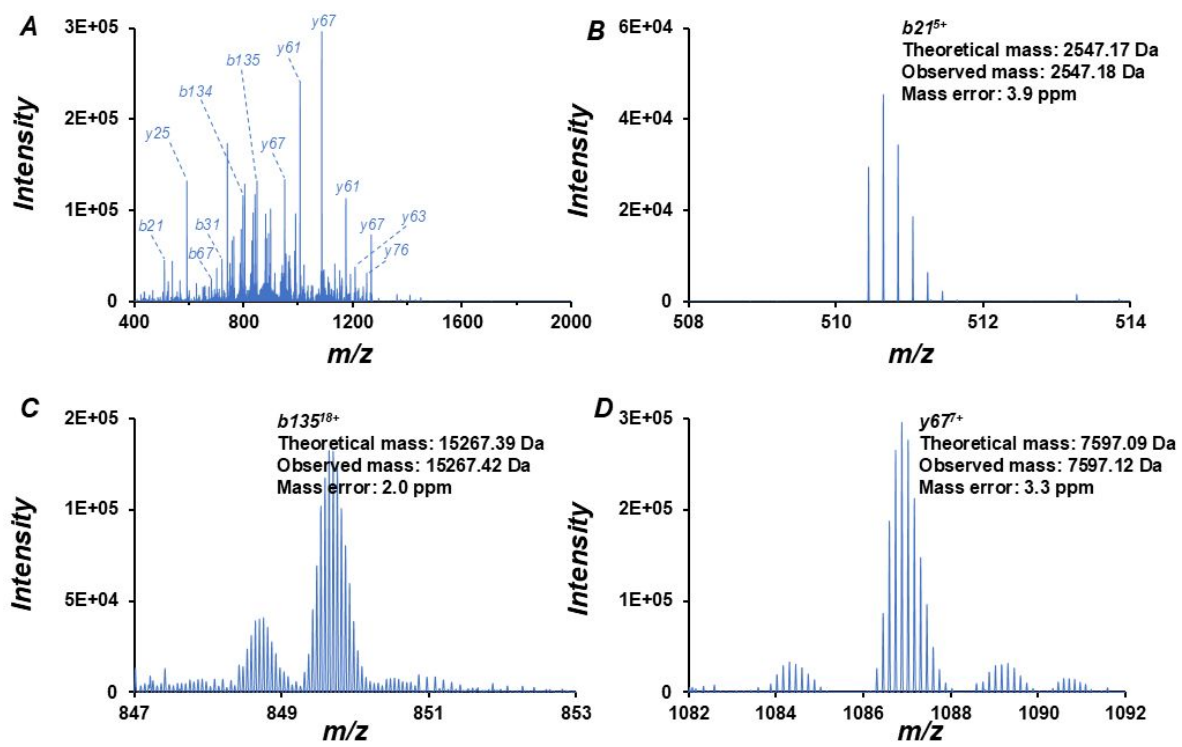

Figure S8. Example annotated MS/MS spectra of CA from HCD. Some high-abundance matched fragment ions are labelled. Isotopic patterns of multiple fragment ions are also shown.

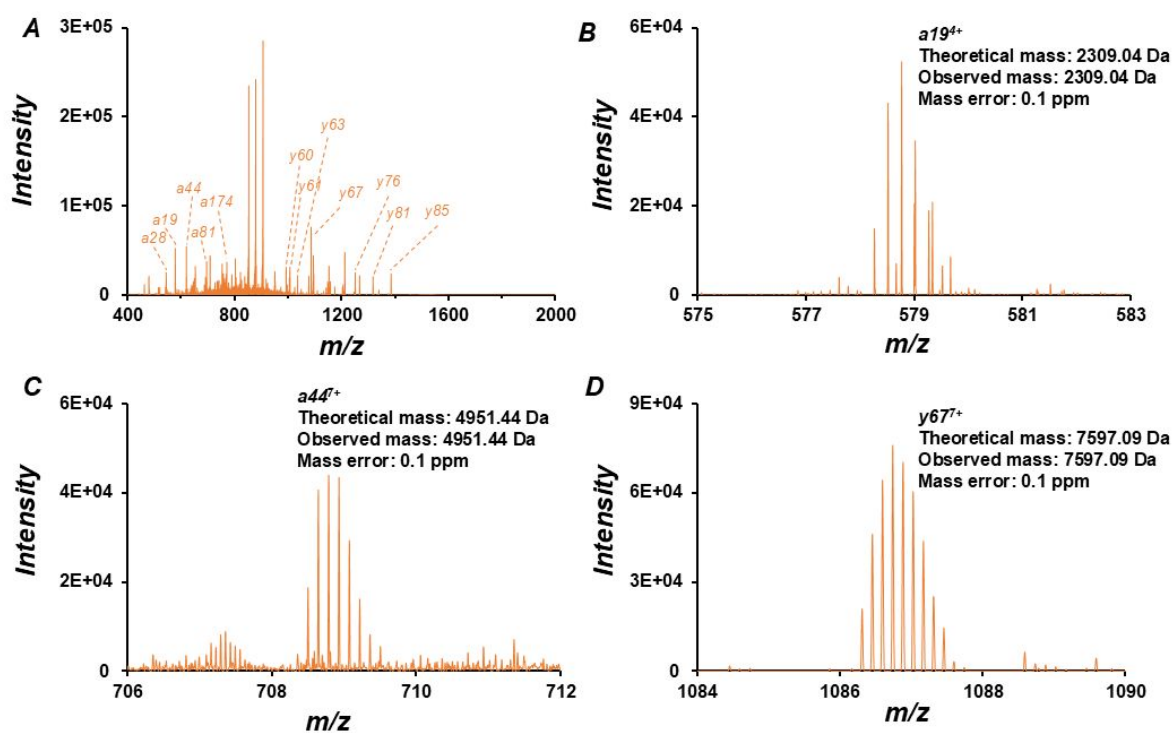

Figure S9. Example annotated MS/MS spectra of CA from UVPD. Some high-abundance matched fragment ions are labelled. Isotopic patterns of multiple fragment ions are also shown.
